# Equitable distribution of SARS-CoV-2 tests 

**DOI:** 10.2471/BLT.21.287398

**Published:** 2022-05-02

**Authors:** Miguel Angel Garcia-Bereguiain, Alfredo Bruno, Diana Morales-Jadan, Jorge E Vidal

**Affiliations:** a One Health Research Group, Universidad de Las Americas, Via a Nayon S/N, 170125 Quito, Ecuador.; b Centro Nacional de Referencia para Influenza y Otros Virus Respiratorios, Instituto Nacional de Salud Pública en Investigación Leopoldo Izquieta Pérez, Guayaquil, Ecuador.; c Department of Microbiology and Immunology, University of Mississippi Medical Center, Jackson, United States of America.

Since its outbreak in 2019, the coronavirus disease 2019 (COVID-19) pandemic, caused by the severe acute respiratory syndrome coronavirus 2 (SARS-CoV-2), has challenged public health systems worldwide.^1^ In a matter of weeks, demand for clinical microbiology diagnosis based on molecular tools increased. The gold standard for the detection of SARS-CoV-2 is the reverse-transcription quantitative polymerase chain reaction (RT-qPCR), although other nucleic acid amplification tests, such as RT-loop-mediated isothermal amplification, are available. Several available RT-qPCR assays (with different sets of primers and probes), developed by public institutions such as the Centers for Disease Control and Prevention in the United States of America or the Charité Hospital in Germany, were endorsed by the World Health Organization (WHO) in the early stages of the pandemic.^2^^,^^3^ The pandemic created a huge laboratory demand and more than 300 commercial nucleic acid amplification tests for SARS-CoV-2 diagnosis became available during the first year of the outbreak. Some of these tests were given emergency use authorization by the US Food and Drug Administration or were included in WHO’s Emergency Use Listing. For others, however, information regarding their clinical performance was scarce and they did not have emergency use authorization in the country of production.^4^^–^^10^ Due to the urgency caused by the novel virus, regulatory agencies adopted more flexible regulatory protocols for the emergency use authorization of SARS-CoV-2 nucleic acid amplification kits. 

Most of the kits are developed by companies from high-income countries. Therefore, many low- and middle-income countries have had to import these diagnostic kits. While some of those assays have obtained emergency use authorization by reputed federal agencies in the countries of manufacture or are included in WHO’s emergency use list, others have been denied local emergency use authorization. In Ecuador, manufacturers of kits not receiving authorization have not disclosed the reasons for the denial.^4^^,^^6^^,^^7^^,^^9^ Because of the high demand for COVID-19 diagnosis, all countries experienced supply shortages, whether of sample collection swabs or nucleic acid amplification kits. Manufacturers gave high-income countries priority access to good quality kits; therefore, some companies selling kits that do not have emergency use authorization at country of origin and are not included in WHO’s emergency use list have targeted markets with inadequate access to quality tests. By taking over the market with those low-quality kits and creating dependency on these kits through extended contracts, manufacturers compromised the access of many of these countries to high-quality COVID-19 diagnostic tools.^6^ Some kits of suboptimal quality manufactured in high-income countries have therefore been distributed in low- and middle-income countries during the pandemic. This practice is unethical because SARS-CoV-2 diagnosis kits should have the same quality standards in all countries. 

In many countries lacking local technology for the detection of SARS-CoV-2 during the global supply shortage of diagnostic kits, public health authorities exempted companies marketing these kits in their countries from proving they had clinical use authorization in the country of production.^4^^,^^6^^,^^7^ These kits might have given false negative testing results, leading to inadequate control of the outbreak. From our experience in Ecuador, no local experimental evaluation of the clinical performance is required before using the assays on patients for the detection of SARS-CoV-2.^6^ Therefore, the health ministry is probably underestimating the countrywide COVID-19 morbidity and mortality.

Two years into the COVID-19 pandemic, important lessons can be learnt to face future pandemics regarding testing capabilities. For instance, public health authorities in low- and middle-income countries could have relied on research conducted by many institutions and universities to assess the performance of SARS-CoV-2 detection assays during the pandemic.^11^^–^^13^ Although the government agency responsible for clinical use authorization in Ecuador lacks funding to carry out proper clinical performance evaluations, Ecuadorian universities and research centres conducted research on clinical performance evaluations on the kits that were available for clinical use in the country. The evaluations showed worrisome differences in clinical performance among commercial kits, with several kits not reaching a reliable sensitivity for an accurate COVID-19 diagnosis. Those kits that were shown to have poor clinical performance lack emergency use authorization at their country of production and/or are not included in WHO’s emergency use list.^4^^–^^10^

Based on these findings, we propose that public health authorities in countries lacking appropriate policies to endorse emergency use authorization for diagnostic tests implement stricter policies. At the minimum, only those kits having emergency use authorization in the country where the manufacturer is headquartered should be allowed, and choice of kits should not be based on price, but on quality. Doing so is particularly relevant for countries such as Ecuador, where local public health authorities do not perform experimental evaluations to grant emergency use authorization to market SARS-CoV-2 nucleic acid amplification kits. Adopting such policies should be encouraged, developed and implemented in-country. Ignoring that inadequate performance assessment of these kits is an issue in many countries could cause unnecessary morbidity and mortality in those countries and in high-income countries, because of imported cases that could lead to new outbreaks. 

The COVID-19 pandemic is one of the most serious public health threats of the last decades and will only be contained through a global health approach. The same is true for future pandemics. This global health approach means that all countries should have diagnostic quality and testing capacities. Ensuring these capacities are available and equally distributed is a human right – that of health – as well as the only way to fight infectious disease outbreaks. This conclusion should be one of the lessons learnt from the COVID-19 pandemic.

We urge public health authorities in low- and middle-income countries to review their protocols for the emergency use authorization of SARS-CoV-2 nucleic acid amplification kits, and revoke authorizations given to under-performing diagnostic kits. We also express our concern to companies exporting low-quality products to low- and middle-income countries and encourage researchers in these countries to contribute with clinical performance evaluation of such detection assays to ensure independent quality control. Finally, we call international public health organizations to act to ensure a fair trade of SARS-CoV-2 tests, based on universal quality standards and without country-income bias.
